# The effect of solution and gel forms of sodium hypochlorite on postoperative pain: a randomized clinical trial

**DOI:** 10.1590/1678-7757-2020-0998

**Published:** 2021-08-11

**Authors:** Esin OZLEK, Hüseyin GUNDUZ, Gizem KADI, Ahmet TAŞAN, Elif AKKOL

**Affiliations:** 1 The University of Van Yuzuncu Yıl Faculty of Dentistry Department of Endodontics Van Turkey The University of Van Yuzuncu Yıl, Faculty of Dentistry, Department of Endodontics, Van, Turkey.; 2 Medicadent Oral and Dental Health Clinic Istanbul Turkey Medicadent Oral and Dental Health Clinic, Istanbul, Turkey.

**Keywords:** Postoperative pain, Root canal irrigants, Root canal preparation, Sodium hypochlorite gel, Sodium hypochlorite solution

## Abstract

**Objectives:**

The aim of this study is to evaluate the effect of using gel and solution forms of NaOCI during the chemomechanical preparation of the root canals on postoperative pain at different time intervals.

**Methodology:**

114 patients with mandibular molar teeth and symptomatic irreversible pulpitis were included in the study. All patients were divided into two groups based on the irrigant used during root canal preparation (n=57): Group 1, 5.25% NaOCI, Group 2, 5.25% NaOCI gel. All groups were filled with gutta-percha and AH Plus root canal sealer using single-cone technique. VAS scale (1-10) was used for postoperative pain assessment. After endodontic treatment, all patients were asked to record their postoperative pain levels at the 6th, 24th, 48th, 72nd hours, and 1 week later. The data were analyzed using Chi-Squared, Independent Samples T, Cochran Q and Friedman tests.

**Results:**

Statistically significant difference was not found between the distributions of pain levels at different times according to the groups (p>0.050). A statistically significant difference was observed between the distributions of pain levels measured at different times in the solution group (p<0.001). A statistically significant difference was found between the distributions of pain levels measured at different times in the gel group (p<0.001). In both groups, highest postoperative pain levels occurred in the first 6 hours. Pain levels of the gel group as 38,5% mild, 17.3% moderate, 5.8% severe and pain levels of the solution group were obtained as 46.2% mild, 26.9% moderate, 9.6% severe at the 6th hour.

**Conclusions:**

The use of the gel form of NaOCI during the chemomechanical preparation of the root canals showed similar postoperative pain when compared to the solution form.

## Introduction

Postoperative pain is a widespread complication after root canal treatments, which is undesirable for patients and physicians.^1^ The incidence of postoperative pain stated in cases is 39% after endodontic treatment and in the first 24 hours, this rate can even be up to 65% and above.^2^ Many mechanical, chemical and microbiological factors play a role in the occurrence of postoperative pain.^3^ It especially appears as a result of extrusion of noninfected debris and solutions into periradicular tissues.^4^ Many studies about this subject exists in the literature.^1^ These studies have focused on different irrigation solutions and activation systems in addition to the mechanical preparation procedures of the root canal.^5 , 6^

Sodium hypochlorite (NaOCI) is the most widely used irrigation solution during root canal treatment.^3^ In addition to its advantages such as antimicrobial activity and organic tissue solvency, however, it also has cytotoxic effects on periradicular tissues. When it is extruded from apical to periradicular tissues during root canal treatment, it damages endothelial cells and fibroblasts, facial nerve palsy, allergic reaction and necrosis may develop.^7^ In a retrospective study conducted by members of the American Association of Endodontists, 42% of clinicians reported that using NaOCI causes postoperative pain or serious complications at least once a year.^8^ That’s why, researchers are in search of a more biocompatible irrigant.

It has been suggested currently that the use of the gel form of NaOCI is a potential option.^9 , 10^ Studies have reported that the solution and gel forms of NaOCI have a similar effect on dentin. In the study of Zand, et al.^10^ (2010) evaluating the smear layer removal activity of the solution and gel forms of NaOCI, and the study of Garcia, et al.^9^ (2013) evaluating the effects of both forms on dentin microhardness, it has been reported that they have similar effects.

The effect of the gel form of NaOCI on postoperative pain has not been studied; therefore, this study compared the effects of using gel and solution forms of 5.25% NaOCI in mandibular molar teeth with symptomatic irreversible pulpitis on postoperative pain. The null hypothesis was that there would be no difference between the gel and solution forms of NaOCI.

## Methodology

### Study design, setting and sampling

**Figure 1:**
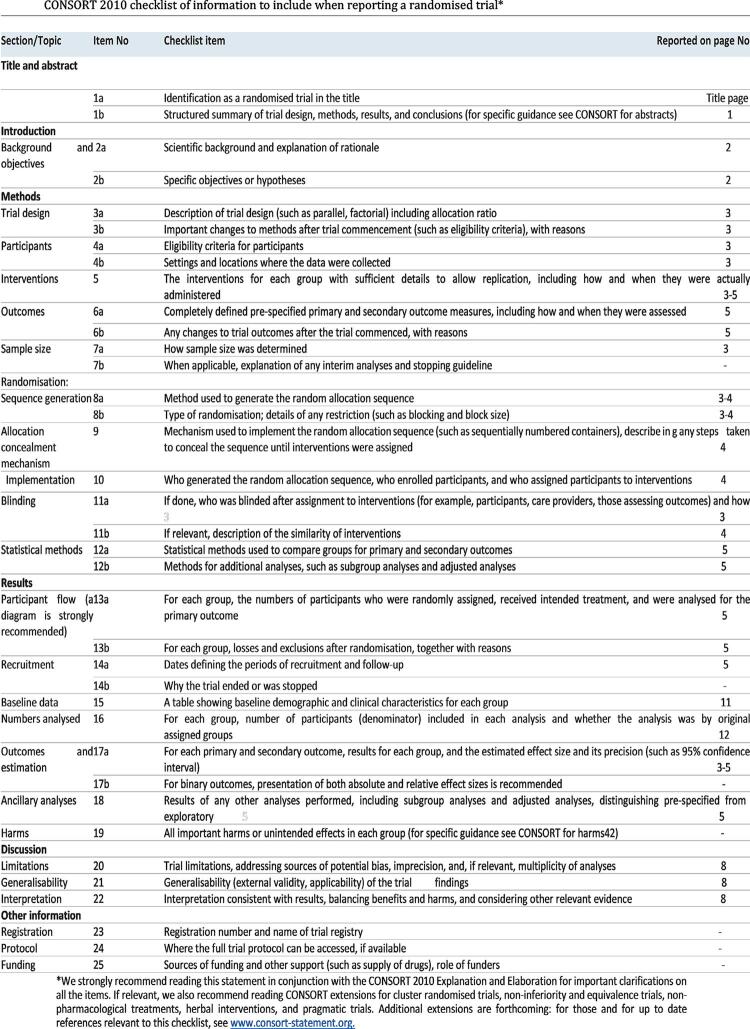
The Consolidated Standards of Reporting Trials checklist

Ethical approval for the study was obtained from the Institutional Review Board and the Ethics Committee of the University. In this clinical trial, Consolidated Standards of Reporting Trials guidelines were followed ( Figure 1 ) and the study protocol was registered on www.clinicaltrial.gov (Identifier: NCT04190355). Participation in the study was voluntary. All patients signed an informed consent form after aims, procedures, benefits and potential risks of the study were explained. The study was conducted by 4 postgraduate students with the same level of experience, trained in endodontic procedures (using rotary instruments, irrigation and canal filling). Power and Sample Size Calculation software version 3.1.2 was used to calculate sample size. With 95% confidence, 95.1% test power and d=0.1121 effect size, the total sample size was determined to be 114.

### Eligibility criteria

The inclusion criteria were as follows:

Healthy individuals aged 18-45 without any systemic disease;

Mandibular molar teeth that were diagnosed with symptomatic irreversible pulpitis that showed prolonged response in the tooth even after the removal of the thermal and electric pulp test;

Teeth with all root canals inclined up to 25° according to Schneider^11^ (1971) method, with two canals in the mesial root and a single canal in the distal root;

Patients with preoperative pain scores between moderate and severe (VAS, 4-10) according to the VAS scale.

The exclusion criteria were the following:

Patients having taken analgesics or anti-inflammatory drugs in the past 12 hours;

Pregnant and lactating patients;

Teeth with radiologically proven periapical lesion;

Teeth that are too damaged to apply rubber dam;

Teeth with resorption, radiological evidence of calcification or open apices;

Patients with traumatic malocclusion;

Patients without occlusal contact.

114 patients were randomized into 2 groups based on the irrigation type during root canal preparation, using a computer program (available at www.randomizer.org). Each group was randomly and equally divided. The allocation ratio was 1:1. In the study, patients were not informed about the division and they were blinded. However, the clinicians could not be blinded due to the nature of the study.

### Treatment procedure

Root canal treatments of all patients were performed in a single session. Dental anesthesia was achieved using local anesthetic solution (Ultracain DS Fort, Hoechst-Marian Roussel, Frankfurt, Germany) containing 4% articaine and 1:200000 epinephrine for inferior alveolar nerve blockade. After rubber dam isolation, endodontic access cavity was prepared using high-speed burs (Dentsply Maillefer, Ballaigues, Switzerland). The working length (WL) was determined using apex locator (Propex Pixi, Dentsply Maillefer) and confirmed to be 0.5-1 mm shorter than the “radiographic apex” by periapical radiographs. The root canals were mechanically prepared using ProTaper Next (Dentsply, Maillefer, Ballaigues, Switzerland) up to X3. All ProTaper Next files were used with an endodontic engine (X-Smart, Dentsply Sirona) at the torque and speed values recommended by the manufacturer. After reaching the WL with size-15 K-type hand file, shaping was continued with brushing motion until the canal length was achieved with X1, X2 and X3 files, respectively. The files were withdrawn at the point where resistance was met before torsional overload occurred and the work continued after the apical opening was checked with size-10 K-type hand file. All patients were divided into two groups based on irrigant used during root canal preparation (n = 57): Group 1, 5.25% NaOCI solution (Imicrly, Konya, Turkey), Group 2, 5.25% Chloraxid gel.

Group 1 (NaOCI solution): The root canals were irrigated with 5 mL of 5.25% NaOCI.

Group 2 (NaOCI gel): Gel form of 5.25% NaOCI (Chloraxid gel, Cercamed, Stalowo Wolo, Poland) was used. After covering the root canal files with NaOCI gel, they were placed in the root canal. During instrumentation canals were irrigated with 5 mL saline.

The irrigation procedure in all groups was performed with a NaviTip irrigation needle (30-G; Ultradent Products Inc, South Jordan, UT), placed 2 mm short of the working length. Once the shaping of the root canals was completed, according to the final irrigation procedure, all canals were irrigated with 5 mL of 17% EDTA solution (Imicryl, Konya, Turkey), 5 mL of 5.25% NaOCI solution and 5 mL of saline, respectively. Root canals were dried with absorbent paper points (Denstply Maillefer, Baillagues, Switzerland). All groups were filled with gutta-percha (Denstply Maillefer, Baillagues, Switzerland) and AH Plus Sealer (Denstply Maillefer, Baillagues, Switzerland) root canal paste in the same session using the single-cone technique. After the quality of obturation was ensured with radiographs, coronal seal was provided with glass ionomer cement (Amalgomer, AHL, Kent, UK). The teeth was restored with composite resin (Filtek Z250, 3M ESPE, St. Paul, Minnesota, USA) and, then, occlusal contacts was checked and relieved where necessary. Each patient was prescribed 400 mg of Ibuprofen and was instructed to take it every 8 hours when felt too severe and extremely unbearable pain to perform his daily activities.

### Postoperative pain assessment

Visual analogue scale (VAS) was used for postoperative pain assessment. After endodontic treatment, all patients were given a detailed form to record their postoperative pain levels at the 6th, 24th, 48th, 72nd hours, and 1 week later. VAS assessment in this form was explained to the patients in detail, and they were asked to mark on the form the pain they felt at the 6th, 24th, 48th, 72nd hours, and 1 week later. One week after the treatment, patients were called by phone. The pain scores that the patients marked on the pain assessment form were learned and recorded in the patient file. According to the values recorded on the VAS, the pain levels were classified as no pain (0), mild pain (1-3), moderate pain (4-6) and severe pain (7-10).

### Statistical analysis

The data were analyzed by using IBM SPSS V23. The compatibility of the quantitative data to normal distribution was examined with the Kolmogorov Smirnov test. Chi-square test was conducted to compare categorical variables according to groups. Independent two sample t-test was performed for normally distributed data in comparison of quantitative variables according to between the two groups. Cochran Q test and Friedman test were used to examine the changes of categorical parameters within the group according to three or more time. Analysis results were presented as mean and standard deviation for quantitative data and as frequency (percentage) for categorical data. A p value of <0.05 was considered statistically significant.

## Results

**Figure 2:**
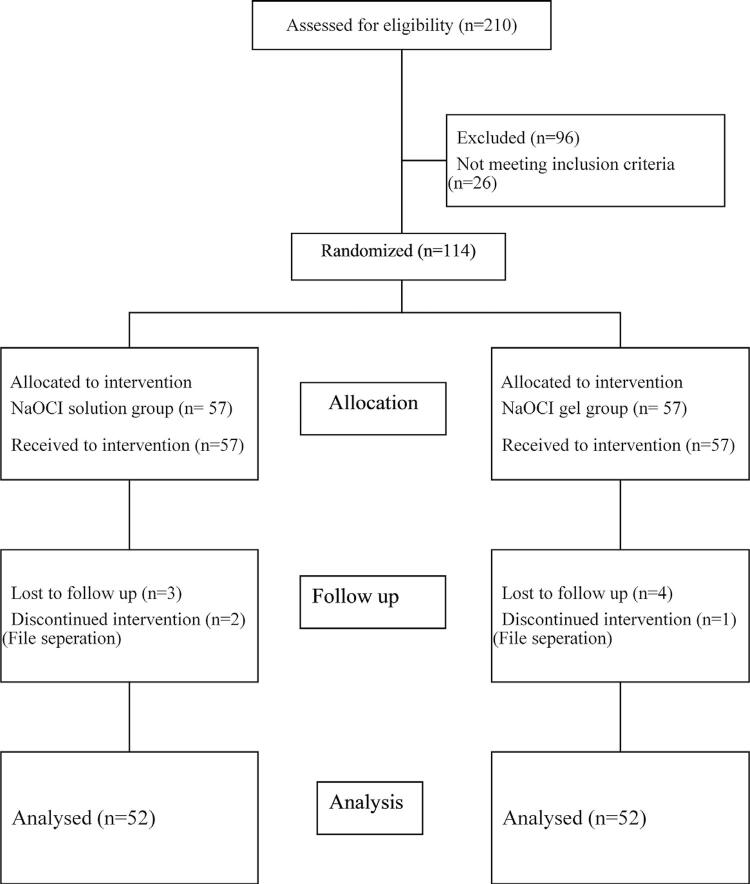
The Consolidated Standards of Reporting Trials 2010 flow diagram

Of the 114 patients who participated in the study, 3 were not included in the analysis, as the instrument was broken in the root canal during root shaping (2 from the solution group and 1 from the gel group) and 7 were not accessible by phone (3 from the solution group and 4 from the gel group) ( Figure 2 ). Demographic data (gender and age) of the groups are shown in Table 1 . No statistically significant difference was found between the groups according to gender distribution (p>0.05).

**Table 1:** Comparison of Age, Gender and Pre-Operative Pain Values by Groups

	NaOCI Solution (n=52)	NaOCI Gel (n=52)	Total	P Value
Age	31.25± 8.76	29,27± 10.32	30.26± 9.58	0.294*
Gender				
Male	24(46.2)	25(48.1)	49(47.1)	0.844**
Female	28(53.8)	27(51.9)	55(52.9)	

NaOCI, Sodium hypochlorite; * Independent samples t test; ** Chi-square test

Statistically significant difference was not observed between the groups according to distributions of pain levels at different times (p>0.05) ( Table 2 ). A statistically significant difference was found between the distributions of pain levels measured at different times in the solution group (p<0.001). A statistically significant difference was observed between the distributions of pain levels measured at different times in the gel group (p<0.001). The mean and standard deviation of pain score of the groups are given in the table 3 .

**Table 2:** Comparison of Pain Levels Between Groups and Within Groups

Time	Pain	NaOCI Solution		NaOCI Gel		Total	P Value *
		n (%)	Pair Wise	n (%)	Pair Wise	n (%)	
Pre-Operative	Mild	7 (13.5)	a	5 (9.6)	a	12 (11.5)	0.813
	Moderate	38 (73.1)		39 (75.0)		77 (74.0)	
	Severe	7 (13.5)		8 (15.4)		15 (14.4)	
6 hour	No pain	9 (17.3)	b	20 (38.5)	b	29 (27.9)	0.106
	Mild	24 (46.2)		20 (38.5)		44 (42.3)	
	Moderate	14 (26.9)		9 (17.3)		23 (22.1)	
	Severe	5 (9.6)		3 (5.8)		8 (7.7)	
24 hour	No pain	21 (40.4)	c	29 (55.8)	c	50 (48.1)	0.219
	Mild	20 (38.5)		17 (32.7)		37 (35.6)	
	Moderate	10 (19.2)		4 (7.7)		14 (13.5)	
	Severe	1 (1.9)		2 (3.8)		3 (2.9)	
48 hour	No pain	29 (55.8)	d	36 (69.2)	d	65 (62.5)	0.556
	Mild	19 (36.5)		13 (25)		32 (30.8)	
	Moderate	3 (5.8)		2 (3.8)		5 (4.8)	
	Severe	1 (1.9)		1 (1.9)		2 (1.9)	
72 hour	No pain	41 (78.8)	e	44 (84.6)	e	85 (81.7)	0.34
	Mild	9 (17.3)		7 (13.5)		16 (15.4)	
	Moderate	2 (3.8)		---		2 (1.9)	
	Severe	---		1 (1.9)		1 (1)	
1 week	No pain	48 (92.3)	f	49 (94.2)	f	97 (93.3)	0.603
	Mild	3 (5.8)		3 (5.8)		6 (5.8)	
	Moderate	1 (1.9)		---		1 (1)	
P Value**		<0.001		<0.001			

NaOCI, Sodium hypochlorite; n, Number of patient;* Chi-square test; **Friedman testi; a-f, There is no difference between times with the same letter.

**Table 3:** The mean and standard deviations of pain score of groups at different time points

	NaOCI Solution	NaOCI Gel
6 hour	2,846±2,388	2,096±2,411
24 hour	1,885±2,111	1,192±2,020
48 hour	0,904±1,432	0,712±1,499
72 hour	0,385±1,051	0,308±0,853
1 week	0,154±0,638	0,077±0,334

NaOCI, Sodium hypochlorite; p = 0.345. No significant differences were found between the groups

## Discussion

The success of endodontic treatment depends on expanding and shaping the root canals, disinfecting and filling them fluid-tight. However, adequate disinfection cannot be achieved with mechanical preparation because of the complex anatomy of the root canals. Debris produced by the instruments during root canal treatment and the unshaped areas left untouched by the canal files affect the success of root canal treatment negatively by acting as a reservoir for microorganisms. Therefore, irrigation solutions should be used during and after the mechanical preparation of the root canals.^12 , 13^ Sodium hypochlorite is the most commonly used irrigant during root canal treatment. However, its cytotoxic effects when extruded into periapical tissues are clinically worrying.^9^ Currently, it has been reported that the use of the gel form rather than the solution form of NaOCI is similarly effective, so the clinical use of the former may be an appropriate alternative to the latter.^10 , 14^

Many studies on postoperative pain levels exists in literature. Most of these studies have focused on different file systems,^15^ single-session and multi-session treatment procedures,^1 , 2^ different irrigation concentrations^16^ and different irrigation activation systems.^5^ Topçuoğlu, et al.^17^ (2017)reported that the level of pain felt by the patients was higher than that of the patients in the manual file group compared to the Nair, et al.^18^ (2018) evaluated the effect of K-Files, Kedo-S and MTwo files on postoperative pain, they found the least pain scores in the MTwo group. Saba, et al.^19^ (2018) reported no difference in the effects of 2% chlorhexidine and 5.25% sodium hypochlorite solutions on postoperative pain. Farzaneh, et al.^20^ (2018) reported that 2.5% NaOCI solution showed lower pain levels in their studies, in which they evaluated the effect of 5.25% NaOCI and 2.5% NaOCI solution on postoperative pain. Moreover, Mostafa, et al.^21^ (2020) also reported that the 1.3% NaOCI solution showed lower pain levels than 5.25% NaOCI. Topçuoğlu, Topçuoğlu and Arslan^22^ (2018) reported in another study that apical positive irrigation (NaviTip) caused more pain than negative apical pressure irrigation system (EndoVac). To our knowledge, the effects of NaOCI’s solution and gel forms on postoperative pain have not been evaluated in any study performed to date. Therefore, the effect of NaOCI’s solution and gel forms on postoperative pain was compared in this study, and the null hypothesis was accepted.

Although no statistically significant difference was found between the distribution of postoperative pain levels of the gel and solution forms of NaOCI (p>0.05), at all times evaluated (6th, 24th, 48th, 72nd hours, and 1 week later), less pain has been observed in the gel group compared to the solution group. As the results show, it can be said that the gel form of NaOCI has a positive effect since its use during the chemomechanical preparation of the root canals caused lower levels of postoperative pain in patients.

Extrusion of dentin particles, necrotic pulp tissue and microorganisms from apical foramen to periapical tissues during chemomechanical preparation of root canals may increase postoperative pain by triggering an inflammatory reaction.^23 , 24^ Therefore, reducing the apical extrusion of debris and solution can reduce the degree of postoperative pain after endodontic treatment. Many studies in the literature evaluating the effect of irrigation techniques on the amount of apical extrusion of debris and irrigation solution exists, and these studies report that more debris is extruded with conventional needle irrigation.^25 - 27^ In addition, many studies in the literature evaluate the effect of irrigation activation techniques on postoperative pain. In these studies, higher levels of postoperative pain were reported in the treatments performed using conventional needles compared to sonic, ultrasonic and laser irrigation, and postoperative pain levels were associated with the amount of debris extrusion.^5 , 28^ Although a lack of evidence is present, we think that lower postoperative pain levels with the use of the gel form of NaOCI in our study may be associated with the amount of apical debris extrusion, and this is in line with studies present in the literature. Further clinical studies are needed to evaluate the effect of the gel form of NaOCI on debris extrusion.

Although the gel form of NaOCI is advantageous to reduce postoperative pain, it is a concern whether the gel form of NaOCI is as effective as the smear removal and disinfection efficiency as the solution form. A limited number of studies focus on this subject in the literature review. Zand, et al.^10^ (2010) They reported that no difference between NaOCI solution and gel forms was observed in terms of smear removal efficiency. Abu Hasna, et al.^29^ (2020) also reported that no difference was found in their studies evaluating the effect on *Enterococcus faecalis* and *Escherichia coli* . Further studies evaluating the smear removal, disinfection and debris extrusion efficiency of NaOCI gel and solution forms are needed.

In our study, a statistically significant difference is present between the distributions of pain levels measured at different times in the gel group (p<0.001). Pain levels of the gel group were obtained as 38,5% mild, 17.3% moderate, 5.8% severe at the 6th hour, 32.7% mild, 7.7% moderate, 3.8% severe at the 24th hour, 25% mild, 3.8% moderate, 1.9% severe at the 48th hour, 13.5% mild, 1.9% severe at the 72nd hour, 5.8% mild 1 week later. A statistically significant difference was observed between the distributions of pain levels measured at different times in the solution group (p<0.001). Pain levels of the solution group were obtained as 46.2% mild, 26.9% moderate, 9.6% severe at the 6th hour, 38.5% mild, 19.2% moderate, 1.9% severe at the 24th hour, 36.5% mild, 5.8% moderate, 1.9% severe at the 48th hour, 17.3% mild, 3.8% moderate at the 72nd hour, 5.8% mild and 1.9% moderate 1 week later. In both groups, highest postoperative pain levels occurred in the first 6 hours, with decrease observed at the following 24th, 48th, 72nd hours and the end of 1 week. The results obtained in this study are consistent with other studies in the literature evaluating postoperative pain periods.^1 , 5 , 6 , 15 , 16^

Studies in the literature report that tooth type in different regions affects postoperative pain. Ali, et al.^30^ (2012) reported more pain in the mandibular region than in the maxillary region, while Arias, et al.^31^ (2009) noted higher incidence of postoperative pain in mandibular molar teeth. This variability in pain may be due to the difference of canal configurations of teeth and their anatomy in the apical third. In this study, mandibular molar teeth were used regarding the studies in the literature.^2 , 6 , 15 , 26^

The painless treatment and post-treatment process provides patients with comfort and physicians with prestige. Therefore, pain research has become increasingly important in all health disciplines in recent years. However, concerns regarding the scientific communication of the sensation of pain exists. Because the sensation of pain is completely relative, varying from person to person.^2^ In previous studies evaluating postoperative pain observed after endodontic treatment, the VAS scale was used because it is simple, valid and reliable.^1 , 3 , 5 , 16^ VAS scale consists of numerical values. This makes it easier for patients to interpret and record their pain perceptions.^1^ In this study, the VAS scale was used to evaluate postoperative pain. Limitation of the present study was the blinding of endodontist was not possible due to the use of solution and gel form of NaOCI.

One of the limitations of this study is that the patients refused to come to the control sessions because root canal treatment was completed in one session. Therefore, pain levels were collected by phone calls. Although the clinicians could not be blinded due to the nature of the study, the researcher who made the phone calls could be blinded about the method of treatment.

## Conclusion

Within the limitations of the study, using gel or solution forms of NaOCI during chemomechanical preparation of root canals, resulted in similar postoperative pain. For both formulations, pain level decreased over time.
